# Theranostic combinatorial drug-loaded coated cubosomes for enhanced targeting and efficacy against cancer cells

**DOI:** 10.1038/s41419-019-2182-0

**Published:** 2020-01-02

**Authors:** Leilei Zhang, Jinlong Li, Dan Tian, Lihua Sun, Xu Wang, Miao Tian

**Affiliations:** 1 Department of Anesthesiology, The Second Hospital of Jilin University, Changchun, China; 2 Department of Gastrointestinal Surgery, The Second Hospital of Jilin University, Changchun, China; 3 Department of Obstetrics and Gynecology, The Second Hospital of Jilin University, 218 Ziqiang Street, Changchun, 130041 China

**Keywords:** Nanostructures, Drug development

## Abstract

Cubosomes, a product of nanobioengineering, are self-structured lipid nanoparticles that act like drug-loaded theranostic probes. Here, we describe a simple method for the preparation of combinatorial drug-loaded cubosomes with, proof-of-principle, therapeutic effect against cancer cells, along with diagnostic capabilities. Anticancer drugs cisplatin and paclitaxel were loaded in the cubosomes in combination. The cubosomes were coated with a layer of poly-Ɛ-lysine, which helped avoid the initial burst release of drug and allowed for a slow and sustained release for better efficacy. Cubosomes were imaged by transmission electron microscope, and their dispersion analyzed in vitro by differential scanning calorimetric and X-ray diffractogram studies. The microscopic images depicted spherical polyangular structures, which are easily distinguishable. The analyses revealed that the drug is uniformly dispersed all through the cubosomes. Further characterization was carried out by zeta-potential measurement, in vitro release, and entrapment efficiency studies. The in vitro studies established that the coating of cubosomes successfully reduced the burst release of drugs initially and confirmed a slow, sustained release over increased time. Comparative cytotoxicity of coated, uncoated, and blank cubosomes was evaluated, using human hepatoma HepG2 cell line, and the formulations were found to be entirely nontoxic, similar to the blank ones. The therapeutic efficiency of the cubosomes against HeLa cells was confirmed by the impedance measurement and fluorescent imaging. Furthermore, the reduction in impedance in cells treated with coated combinatorial cubosomes proved the impairment of HeLa cells, as confirmed by fluorescence microscopy.

## Introduction

The main aim of nanobioengineering is to improve the already available theranostic tools in medical sciences. Theranostics is a wide field in biomedical engineering involving in vitro diagnostics and prognosis. It involves in vivo imaging of molecules and cell, therapeutics in molecular medicine, image-guided microscopy and therapeutics, biosensors, system biology, and transcriptional medicine and even treatments which are customized or personalized. Therefore, a huge number of hard and soft nanocarriers encapsulating drugs or imaging dyes have been designed and applied for various purposes^1^. Hard nanocarriers comprised metallic or polymeric nanoparticles^2–6^, whereas soft nanocarriers include liposomes^7–9^, carbon tubes, quantum dots, dendrimers^10,11^, and many more.

Theranostic nanobioengineering boasts of various nanocarriers, among which cubosomes are the most recent. Cubosomes are self-structured, liquid crystalline lipid nanoparticles^12^, which are derived from bulk bicontinuous lipid cubic-phase steadied by pluronics^13,14^. The bulk bicontinuous lipid phase is organized in a three-dimensional honeycomb structures. They have cavern-like structure, which may lead to efficient drug loading. Hence, there have been reports of cubosomes acting as efficient drug carriers topically or subcutaneously^15,16^. Murgia et al.^17^ have reported theranostic probes, which are actually drug-loaded customized fluorescent probes. Cubosomes coated with polyelectrolytes have been demonstrated that prevents the initial burst release of drug^18^. Murgia et al.^17^ have also extended their work to target cancer. Cancer is the second leading cause of mortality after myocardial infarction^19^. The primary requisite for cancer treatment is efficient and targeted delivery of drug to the tumor site. Therefore, cubosomes owing to their small size may lead to improved permeation and retention at the site of cancer. Cubosomes have an added advantage over the liposomes in possessing greater volume for accommodation of increased quantities for drug resulting in better payload, less viscosity^20^, and a hydrophobic core^21^. They possess bicontinuous channels of water that bypass the core, and hence these stealth characteristics make them extremely effective for delivery of drug. Recently, the cubosomes or bicontinuous cubic crystalline nanocarriers have been studied in detail for their theranostic applications^19,22,23^. These may be altered as per the need, they may be coated or surface-modified to avoid phagocytosis, or may be layered to avoid excess release of drugs^24^. It has been reported that these bicontinuous cubic crystalline nanocarriers may be administered via oral, percutaneous, or parenteral routes^25^. The only disadvantage of the cubosomes lies in the retention of hydrophilic drugs, because of the nature of its core.

Cubosomes have acted as nanovehicles for vaccine antigens^26^ and anticancer drugs such as 5-fluorouracil^25^ and cisplatin^27^. But a combination of drugs loaded in cubosomes for targeting cancer cell line has not been reported before. It has been reported that there exists an extremely high mortality rate in malignancies occurring in the genital tract of the female which includes cervical, ovarian, and endometrial cancers. This may result due to recurrence of cancer after persistence of minute residue of malignancy in the cells^28^. Therefore, in this study, we prepared coated cubosomes decorated with two hydrophobic anticancer drugs cisplatin and paclitaxel to study their efficacy against the HeLa cells, the most documented cell line of cervical cancer. Our work describes a simple method of preparation of cubosomes which may be effectively extrapolated to large-scale manufacturing, if needed. Cisplatin is rendered as one of the most effective anticancer drug, especially against several types of tumors. Paclitaxel is also reported to be a potent chemotherapeutic drug which causes torn membranes in HeLa cells^29,30^. It has been seen that cancer cells develop an ability of survival against a wide range of anticancer drugs, including cisplatin, doxorubicin, vinblastine, paclitaxel etc. This ability of cancer cells is being enhanced by decreasing the drug absorption in the cells by releasing the drug outside the cells. This is achieved by altered structure of membrane transporters reducing the drug transport. Also, mutations may occur decreasing the number of the transporters, thereby reducing the drug absorption significantly^26^. Hence, encapsulation of a combination of these drugs in an in vitro nanocarrier manufactured outside the body and transport to the site of action seemed to be a better and efficient mechanism. The cubosomes were coated with a layer of poly-Ɛ-lysine to avoid the initial burst release of the drug and allow a slow, sustained release for better efficacy. The particular cell line used in this study is well documented one.

## Materials and methods

### Materials

RYLO MG 20, a commercial grade monoolein emulsifier, comprised predominantly glycerol monoleate (total monoglyceride >95% and bi-, tri-glycerides, and free glycerol <1%), was obtained from Danisco Corporation Ltd. (India). Poloxamer 407, paclitaxel, cisplatin, and poly-Ɛ-lysine were obtained from Sigma-Aldrich (Beijing, China). For each experiment, milli-Q water was utilized. Analytical grade chemicals were obtained from Sigma-Aldrich.

### Cell culture

Human hepatoma HepG2 cell line was acquired from ATCC China. The cells were cultivated in RPMI 1640 at 37 °C with 5% CO_2_ in a moist environment. RPMI was used as per Nasr et al.^25^, instead of the regular DMEM media. RPMI 1640 was enriched with 10% fetal bovine serum for cytotoxicity assays along with 2 mM L-glutamine, 50 UI/mL penicillin, and 50 UI/mL streptomycin^31,32^. After every 3 days, the medium was replaced. Cell cultures which have attained confluency were dislodged using trypsin, and the cells were planted into 96-well plates at a density of 10^6^ cells/ml. Before being exposed to cubosomes, the cultures were retained at 37 °C for 24 h to allow the cells to achieve confluency and attach to the well plates. These cultured cells were utilized for cytotoxicity and impedance studies.

Human cervical cancer cells HeLa (obtained from ATCC, China), for the fluorescent studies, were obtained from ATCC and cultured in a combination of Eagle’s minimum essential medium (Sigma-Aldrich, China) with 10% fetal bovine serum in an incubator with humidified air or 5% CO_2_ at 37 °C. Every 3 days media was renewed, and when reaching 90% confluence cells were passed.

### Preparation of blank and drug-loaded cubosomic gel

For the preparation of cubosomic gels, the procedure was adapted from Nasr et al.^25^ with necessary modifications. The procedure is being detailed here. For blank cubosomes, in a water bath 2.25 gm of RYLO and 0.25 gm of poloxamer 407 were melted at 70 °C. The molten solution and 4 mL of deionized water (70 °C) were mixed dropwise by vortexing at high speed at room temperature to accomplish state of homogeneity. For 48 h, continuously equilibration of the mixture was implemented at room temperature to obtain the cubosomic gel. For drug loading, before the addition of the molten mixture of RYLO and poloxamer 407, 25 mg of each cisplatin and paclitaxel was added to 4 ml of deionized water and mixed thoroughly. The rest of the methodology was followed exactly as that of the blank cubosomic gels. The cubosomic gels were kept at ambient temperature for further processing. For preparation of individual cisplatin and paclitaxel cubosomic gels, 50 mg of the drug was mixed with deionized water, and rest of procedure was duly carried out as mentioned above. The cubosomes were named as follows: cisplatin-loaded cubosomes (CIS), paclitaxel-loaded cubosomes (PAX), and dual drug-loaded cubosomes (DUAL).

### Preparation of cubosomes

To prepare the cubosomes, the cubosomic gels were dispersed in 20 ml of deionized water followed by vortexing at high speed for 5 min. Coating of the cubosomes was carried out in a simple manner modified from Deshpande et al.^12^. The estimated density and surface area of cubosomes were measured in water, and dropwise premeditated amount poly-Ɛ-lysine was added under continuous vortexing. Particular emphasis was laid on adding poly-Ɛ-lysine slowly so as not to cause phase separation of cubosomes. Samples containing uncoated CIS, PAX, and DUAL were also kept for comparison during studies.

### Morphological analysis of the cubosomes

Morphological analysis of the coated, uncoated drug loaded, and blank cubosomes was done using a high-resolution TEM, JEM-2010HR microscope fortified with twin lens, the electron source being LaB6 and power at 80 kV. On a 200-mesh carbon-coated copper grid, a droplet of cubosome was placed carefully, and the excess droplet was soaked up using an absorbent filter paper. Staining of the samples was done with 1% sodium phosphotungstate solution, and using magnification up to ×1,000,000 the cubosomes were viewed. The samples were dried in air and incubated overnight, and after 12 h the TEM images were viewed.

### Zeta-potential measurement

The size of the cubosomes and zeta-potential values were attained by laser-Doppler anemometry using a Zetasizer Nano Series System (Malvern Instruments Ltd). About 1 ml of the blank cubosomes, CIS, PAX, and DUAL (coated and uncoated) was apportioned to the zeta cell following which measurements were taken. The experiment was performed at 25 °C. The Zeta-potential values were calculated as the average of three measurements of the values of electrophoretic mobility.

### Entrapment efficiency

Ultrafiltration centrifugation was done to calculate the drug entrapment efficiency of the cubosomes^33^. In all, 1 mL of freshly made CIS, PAX, and DUAL (coated and uncoated) was diluted with deionized water to make the volume 10 mL. Next, 3 ml of diluted CIS, PAX, and DUAL (coated and uncoated) was retained in centrifuge tubes (Millipore, USA), and the diluted samples were centrifuged for 15 min at 4000 rpm. The ultrafiltration membranes were of diameter 62 mm (Amicon, YM series) and may be a place for retention of small amount of drugs^34^, hence adsorption of the drug therein was examined by separation of known concentrations of drug solution through the membrane and consequently assessing the concentration of the drug in the filtrate. CIS, PAX, and DUAL present in the filtrate was measured spectrophotometrically. The amounts of entrapped CIS, PAX, and DUAL were obtained by deducting the quantity of free drug from the total drug assimilated in 1 mL of cubosomal solution. Determination of the total amount of CIS, PAX, and DUAL assimilated in 1 mL cubosomal solution was done after adding 9.0 mL of methanol to dissolve CIS, PAX, and DUAL cubosomes. CIS, PAX, and DUAL drugs were assayed spectrophotometrically in the resultant solution taking methanol as blank. The entrapment efficiency (EE) was determined as per the following calculation:

$${\mathrm{Encapsulation}}\,{\mathrm{efficiency}}\,(\%)\, {=} \,{\mathrm{weight}}\,{\mathrm{of}}\,{\mathrm{drug}}\,{\mathrm{in}}\,{\mathrm{cubosomes}} \,{\div}\, {\mathrm{weight}}\,{\mathrm{of}}\,{\mathrm{drug}}\,{\mathrm{originally}}\,{\mathrm{added}}\, {\times}\, {100}$$

### X-ray diffraction (XRD) assay

XRD patterns were obtained of CIS, PAX, and DUAL (coated and uncoated) as well as free cisplatin, paclitaxel, and blank cubosomes with the help of X-ray diffractometer (X'Pert-PRO Diffractometer, PANalytical, The Netherlands). Recording of the diffractograms were done in the following settings: 45 kV voltage, 30 mA current, 0.021 steps, and the counting rate 0.5 s/step. These statistics were recorded at room temperature. Scattering angle (2θ) ranged 4–50° was used to collect the figures.

### Differential scanning calorimetry (DSC)

DSC was performed on CIS, PAX, and DUAL (coated and uncoated) as well as free cisplatin, paclitaxel, and blank cubosomes using a thermal analysis system (DSC-60, Shimadzu, Japan). An aluminium pan was kept under a nitrogen atmosphere on which 5 mg of each samples were heated at a continuous rate of 10 °C/min. For reference, a comparable empty pan was utilized. The process was adapted from Nasr et al.^25^.

### in vitro release studies

Suitable modifications were made in the procedure adapted from Gupta et al.^35^, and the dynamic dialysis method was done. A dialysis bag of 3 ml was utilized for the procedure, which had a molecular weight cutoff of 7000 g/ml. In total, 2.5 ml of each CIS, PAX, and DUAL (coated and uncoated) were loaded in the dialysis bag. Enormous molecules, which remained undissolved, were drawn out through the dialysis bag to confirm adequate media for un-hindered dissolution. The dialysis bag is depicted in Fig. 5a. The dialysis bag was retained in 2.5 L of 10 mM pH 7.4 phosphate buffer solution and to confirm sink conditions, the buffer was replaced every 3 h. Throughout the experiment, the temperature of the buffer was kept at 37 °C. Continuous withdrawal of 150 µl of solution with replacement by fresh buffer of the same amount at regular intervals of 4, 12, 24, 36, 48, and 72 h was done. Utilizing UV–Vis spectroscopy, the concentration of drug in the samples was seen at 266 nm and then equated with standard curve fitting. The dialysis was complete once there was no drug detected in the solution. Measurements were done in triplicates.

### Cytotoxicity assay

Cytotoxicity of control (PBS), blank cubosomes, CIS, PAX, and DUAL (coated and uncoated) was evaluated by MTT assay as per Namdeo et al.^31^ with necessary modifications. Following the procedure mentioned above, confluent cell cultures were obtained. Harvesting and gestation of the cells were carried out in 96-well plates at the concentration of 2 × 10^3^ cells/well. Gestation of cells was carried out for 72 h to ensure viable cells. To the viable cells, the following were supplemented 0.1 mL of PBS (control), blank cubosomes (0.1 mg/ml), CIS (0.1 mg/ml), PAX (0.1 mg/ml), and DUAL (0.1 mg/ml), and again gestated for 48 h. Biological drugs are generally formulated at 0.1 mg/ml, hence the cubosomal dosage was decided as 0.1 mg/ml. Then, 20 μL of MTT solution (5 mg/mL) was supplemented in each well, and incubation was carried out at 37 °C for 4 h. Following incubation, the absorbance was measured at 492 nm using a microplate reader. The results were reiterated for five times and expressed as mean ± SD.

Using live/dead cell viability assay kits, cytocompatibility of control (PBS), blank cubosomes, CIS, PAX, and DUAL was also assessed. Live/dead cell assay kit was obtained from Thermo Fisher Scientific, China. As per the instructions on the kit by the manufacturer, the procedure was carried out.

### Impedance measurements

The ECIS (electric cell–substrate impedance sensing) device is made up of eight wells, and current flow through the solution was detected by ten gold microelectrodes present in each well (250 μm diameters). The volume of each well was 600 μl with the substrate area being 0.8 cm^2^. RPMI 1640 media was utilized to incubate the ECIS devices overnight within a tissue culture incubator. HeLa cell cultures which have attained confluency were obtained using following procedure. Cells were incubated for 72 h and the following were added: empty cubosomes (0.1 ml), CIS (0.1 ml), PAX (0.1 ml), and DUAL (0.1 ml) (coated and uncoated). After additional incubation period of 48 h, within a frequency range from 100 Hz to 1 MHz in a logarithmic scale, the impedances of the samples were measured.. Subsequently, the impedance data were incorporated into ZsimpWin (Ver. 3.10) to fit it. The equivalent circuit was acquired from a study by Pradhan et al.^36^. RE and RI represent the solution resistance and charge transfer resistance, while CS and QM are the capacitance of water and interface impedance of cells.

### Statistical analysis

Entire data in this study were expressed as means and standard deviation (mean ± SD), and processed by Origin 8. Calculations were accomplished by means of the one-way analysis of variance (ANOVA). The difference was considered to be statistically significant, if *P* < 0.05. All analysis and investigations in impedance studies were carried out in triplicates to confirm reproducibility, and the data were represented with their equivalent relative standard deviations (RSD).

### Fluorescent studies with HeLa cells

In vitro cultures of human cells may deliver improved understanding of the reactions of a particular cell type to specific drugs. Following the procedure mentioned above, confluent HeLa cell cultures were obtained.

Nanoformulations of (coated and uncoated) cubosomes (CIS, PAX, and DUAL) were added to the cell culture at the ratio of 1:1000 as was demonstrated by Murgia et al.^17^ (2 µl of cubosomal solutions were added to 2 ml of media containing cells). Cell Explorer™ fluorescence imaging kits (Biochem Life Sciences, India) was used for staining the HeLa cells. This specific kit is intended to consistently stain live HeLa cells in green fluorescence for comparatively extended time. The cells were fixed to preserve the pattern of imaging. The kit utilizes a nonfluorescent dye that becomes intensely fluorescent upon entering into live cells. This gives the live HeLa cells a stable fluorescence signal for moderately lengthier time period. This dye consists of hydrophobic molecules which easily permeates live cells. Observations were made using a Zeiss (Axioskop) upright microscope (Zeiss, Germany). Cellular fluorescence intensity was determined by ImageJ software. The fluorescent intensity of ten cells in the image were measured, and mean was calculated to determine mean fluorescent intensity.

## Results

### Preparation of cubosomes

The process of mechanical stirring has been utilized to prepare the blank, and the CIS, PAX, and DUAL cubosomal formulation. The cubic gel phase of RYLO and water was dispersed using poloxamer 407 as a stabilizer. There were no aggregates as result of uniform stirring. The cubosomal nanoformulation appeared as an opaque white gel with no lumps in it. The ratio of RYLO; water was maintained as 9:1 w/w as suggested by Nasr et al.^25^ who clearly demonstrated that cubosomal nanoformulation made in this ratio had unique characteristics which made adsorption of hydrophobic drugs easier.

### Morphological analysis

**Fig. 1:**
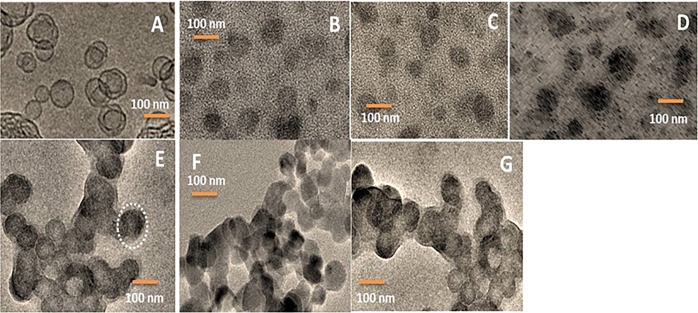
TEM images for morphological analysis of cubosomes. **a** Blank cubosomes. **b** Uncoated CIS. **c** Uncoated PAX. **d** Uncoated DUAL. **e** Coated CIS. **f** Coated PAX. **g** Coated DUAL. Note the spread out polyangular edges of the uncoated cubosomes and rigid structures of the coated ones.

Morphological analysis of the CIS, TAX, and DUAL (coated and uncoated) cubosomes was done by transmission electron microscopy, shown in Fig. 1. The blank cubosomes were cubical in structure unlike the drug-loaded ones (both coated and uncoated), which have become spherical with a lot of polyangular edges. The inner structure of the blank cubosomes was similar to cubic structure. The cavernous structure indicated in the blank cubosomes was the site where the drug may be localized for release. There are also evidences of internal microstructure as depicted in drug-loaded cubosomes. However, the shapes are unique and are easily distinguishable. Some internalized vesicles are also seen which may probably have originated due to application of agitation or repeated stirring. However, the number of vesicles is almost negligible.

### Size, Zeta potential, and polydispersity index (PDI) measurement

Measurement of size of the cubosomes and zeta potential may provide a physiological insight into their functions in milieu. Following integration in the HeLa cell line, size and zeta-potential administrate the behavior of the CIS, PAX, and DUAL (coated and uncoated) and subsequent drug release after the normal physiological distegration of cubosomes. These factors may also govern the toxicity of the cubosomes and the targeted drug delivery to specific tissues. Furthermore, these may also influence drug loading, release, and stability inside cubosomal nanoformulations^37^. The functional performance of the CIS, PAX, and DUAL is governed by the physicochemical properties, including size and charge^38^. Hence, the measurement of zeta potential was carried out. The sizes and zeta potential of the cubosomes are shown in Table 1.

**Table 1:** Sizes, PDI, and zeta potential of cubosomes.

	Size of the cubosomes (nm)	Polydispersity index (PDI)	Zeta potential (mV)
Blank cubosomes	90 ± 3	0.17 ± 0.01	−24.5 ± 0.3
CIS uncoated	102 ± 4	0.14 ± 0.01	−22.4 ± 0.4
CIS coated	103 ± 2	0.15 ± 0.01	−2.8 ± 0.1
PAX uncoated	101 ± 4	0.13 ± 0.03	−23.1 ± 0.1
PAX coated	102 ± 3	0.12 ± 0.01	−2.9 ± 0.1
DUAL uncoated	115 ± 2	0.11 ± 0.02	−27.4 ± 0.2
DUAL coated	119 ± 4	0.11 ± 0.01	−3.4 ± 0.1

Mean particle sizes of blank cubosomes are 90 ± 3 nm, whereas the uncoated CIS, PAX, and DUAL are 102 ± 4, 101 ± 4, and 115 ± 2, respectively, followed by coated CIS, PAX, and DUAL being 103 ± 2, 102 ± 3, and 117 ± 4, respectively. The higher particle sizes of the DUAL are due to the dual-drug loading. The coating caused an added increase in the sizes too. However, a trend of high particle size is seen in all the cubosomal nanoformulations. This may be an indication of increased use of higher concentration of poloxamer 407 (10%) as a stabilizer. The use of higher concentration of poloxamer 407 may act to stabilize the cubic gel phase and prevent them from aggregating, but it also promotes formation of vesicles, which is not much desirable^39^. Negative zeta-potential originates as a result of absorption of hydroxyl ions on the surface of the cubosomes^40^. RYLO used in this study is known to possess free oleic acid which may attribute to the high zeta potential of blank cubosomes and decreased as the absorption of drugs in the uncoated cubosomes increased. However, in the coated cubosomes, the zeta-potential dropped down to −2.8 ± 0.1, −2.9 ± 0.1, and −3.4 ± 0.1 in CIS, PAX, and DUAL, respectively, providing evidence of complexation of the surface of cubosomes due to coating.

### Entrapment efficiency (EE %)

**Fig. 2:**
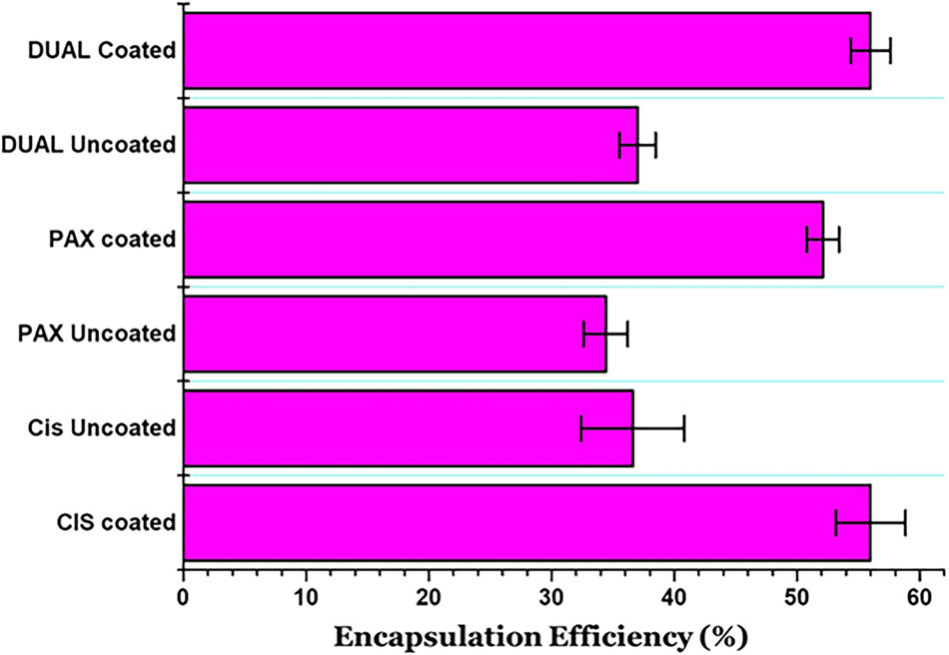
Entrapment efficiency of the coated and uncoated CIS, PAX, and DUAL cubosomes.

The entrapment efficiency of the coated and uncoated CIS, PAX, and DUAL are depicted in Fig. 2. There is much dissimilarity between the coated and uncoated CIS, PAX, and DUAL as seen. The EE % of coated and uncoated CIS was found to be (56 ± 2.83)% and (36 ± 4.28)%, respectively. The EE % of coated and uncoated PAX was found to be (52 ± 1.33)% and (34 ± 1.88)%. The EE % of coated and uncoated DUAL was found to be (56 ± 1.63)% and (37 ± 1.48)%, respectively. Low EE % in uncoated ones may be due to the fact that the drugs loaded were hydrophobic and extremely mobile in nature. They do not associate with the water content in the cubosomes where there is large water retention and hence they may be leaking out through the aqueous channels in the surrounding deionized water. Their retention in the aqueous channels is also very transitory because of the hydrophobic nature. But after a layer of coating of poly-Ɛ-lysine, there is a visible difference in the EE %, indicating reduced amount of drug leakage through the channels since poly-Ɛ-lysine forms a protective coat around the inner core. There were also previous reports of the lipophilic drugs being leaked out from cubosomes during the ultrafiltration and centrifugation processes, because of which we aimed toward coated cubosomes^41^.

### X-ray diffraction studies

**Fig. 3:**
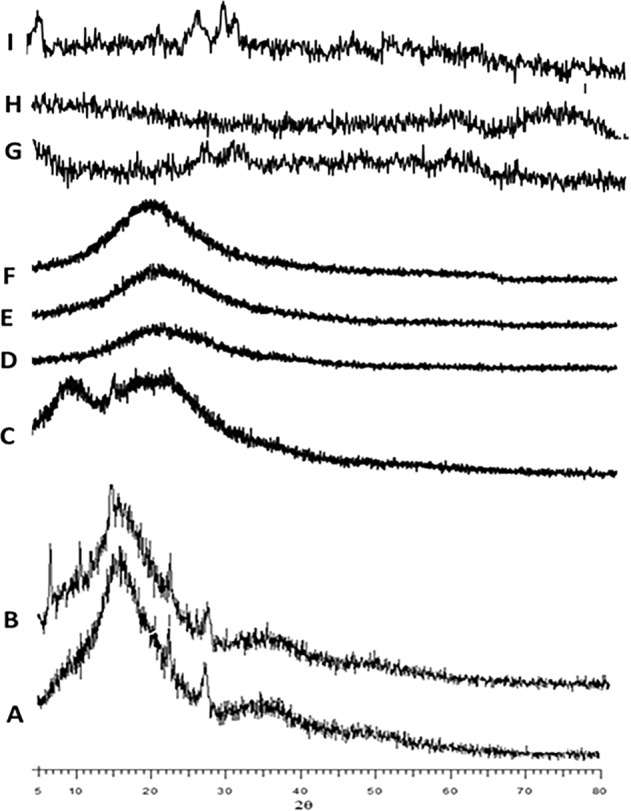
X-ray diffractograms of blank, free cisplatin, free paclitaxel, coated and uncoated CIS, PAX, and DUAL cubosomes.

XRD studies were carried out to understand the physical attribute of drugs in the decorated cubosomes indicated in Fig. 3. Wherein the free drug, we see characteristic reflection of crystalline nature of drugs, this difference virtually vanishes in coated, uncoated, and blank cubosomes. It is interesting to note that even there is not any difference in the diffractograms of coated and blank cubosomes. This is a clear indication of the fact that the drugs have been uniformly dispersed all through the cubosomes and existed in a noncrystalline phase.

### DSC studies

**Fig. 4:**
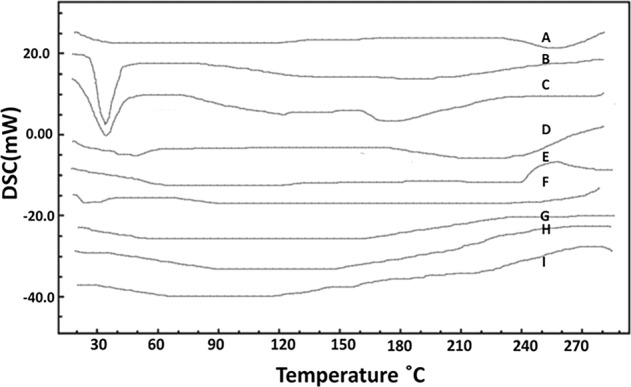
DSC graphs of blank, free cisplatin, free paclitaxel, coated and uncoated CIS, PAX, and DUAL cubosomes.

In the DSC thermograms as seen in Fig. 4, a similar trend to that of XRD is seen. The free cisplatin and paclitaxel exist in crystalline forms showing sharp peaks, whereas in the coated and uncoated cubosomes, these are virtually not seen. These indicated that the drugs were not present in the cubosomes in crystalline form, but dispersed all throughout the cubosomes. Hence, the sharp peaks of the crystalline forms have disappeared from the thermogram graph. These were a reminder that the drugs existed in cubosomes in noncrystalline phase.

### In vitro release studies

**Fig. 5:**
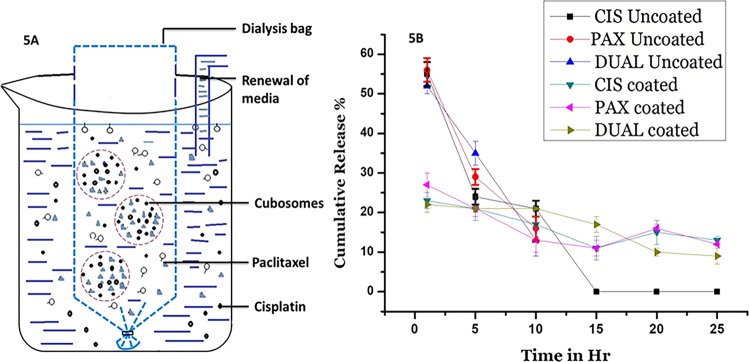
In vitro drug release studies. **a** Graphic adaptation of the dialysis bag for the in vitro release studies. **b** In vitro release of drugs from coated and uncoated CIS, PAX, and DUAL cubosomes.

A graphic adaptation of dialysis bag is shown in Fig. 5a. The results of in vitro release studies are illustrated in Fig. 5b. They show an expected but interesting trend. In the uncoated CIS, PAX, and DUAL, there is a huge initial burst release of drug after a tie period of 1 h. In uncoated CIS, 55 ± 3% of total drug was released, which was 52 ± 2% and 56 ± 3% in case of uncoated DUAL and PAX, respectively. This was followed by a slow release after a time period of 6 h, and no release after 10 h. These were the drugs that were weakly incorporated in the cubosomes or in the aqueous channels bypassing the cubosomes. The cubosomes had a relatively larger surface area as was seen in the TEM images which also made the drugs more mobile. Moreover, the hydrophobic nature of drugs also made them eject faster from the cubosomes which were hydrophilic in nature. The burst release of the drugs has been reported by several studies^41,42^. On the other hand in coated CIS, PAX, and DUAL, the initial release of drugs after 1 h was extremely low. The percent of initial drugs release within an hour in CIS, PAX, and DUAL was 23 ± 3%, 27 ± 2%, and 22 ± 3%, respectively. But a slow and steady drug release continued upto 25 h. This may be attributed to the single-layer coating of the poly-Ɛ-lysine, which restricts the diffusion of the drugs initially. It has already been reported that in cubosomes diffusion is the main mechanism of release^25^. Furthermore, the aqueous channels may have turned to be narrow and tortuous as a result of the coating, which may be the reason the drugs are not allowed to travel freely as they had been doing in the uncoated ones. This study reinforces the fact that cubosomes provides a slow, steady, and efficient matrix for release of drugs that may be utilized for theranostic purposes.

### In vitro cytotoxicity studies

**Fig. 6:**
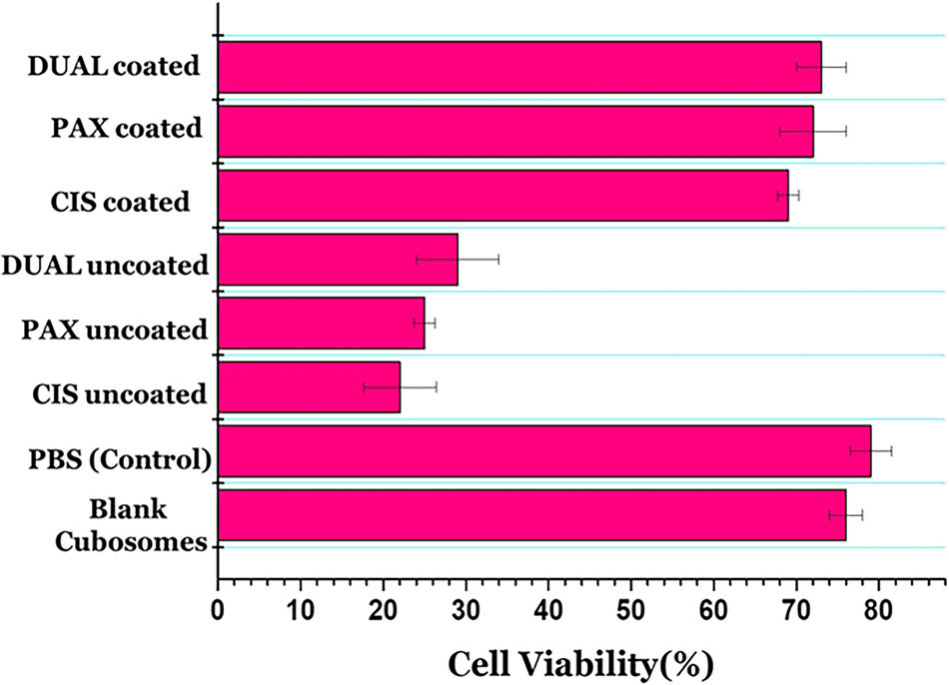
In vitro cytotoxicity was evaluated in cubosomal-coated and -uncoated CIS, PAX, and DUAL formulations.

**Fig. 7:**
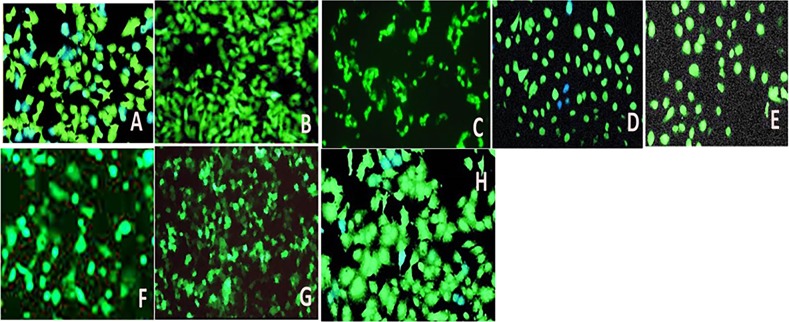
Cell viability studies on exposure of Human hepatoma HepG2 cells to PBS (control) blank, coated and uncoated CIS, PAX, and DUAL cubosomes.

Cytotoxicity was evaluated in cubosomal-coated and -uncoated formulations, and the results are depicted in Fig. 6. The cytotoxic effects of free cisplatin and paclitaxel were also seen in human hepatoma HepG2 cell line. It was clearly seen that free cisplatin and paclitaxel were comparatively toxic to HepG2 cells than the cubosomes. Whereas cytotoxicity of the uncoated ones were more than the coated ones may be attributed to the faster release of drugs in case of the uncoated ones. The results may be related to the in vitro release studies. The weak association of the drugs with the cubosomes in the uncoated ones and depositions in the aqueous channels may cause release of greater amount of drugs, which may have the potential toxic effects on cells. One of the major reasons of encasing the drugs in the cubosomes was to potentially reduce their toxicity along with avoiding burst release. The increased viability of the cells in case of the coated cubosomal formulations is a testimony to the same, as illustrated in Fig. 7. The cell viability in case of PBS-treated cell is 79%. The cells viability in case of blank cubosomes is 76%, which decreased to 22, 25, and 29% in case of uncoated CIS, PAX, and DUAL, respectively. The cell viability increased to 69, 72, and 73% in coated CIS, PAX, and DUAL cubosomes, which is almost comparable with the blank ones. This also illustrates that the coating of poly-Ɛ-lysine was protective both for the cells and well as unnecessary release of large amounts of drugs causing potentially toxic effects and cell death. Blank cubosomes did not have any interactions with the cells which mean the drugs weakly embedded in them have cytotoxic effects.

### Impedance measurements

**Fig. 8:**
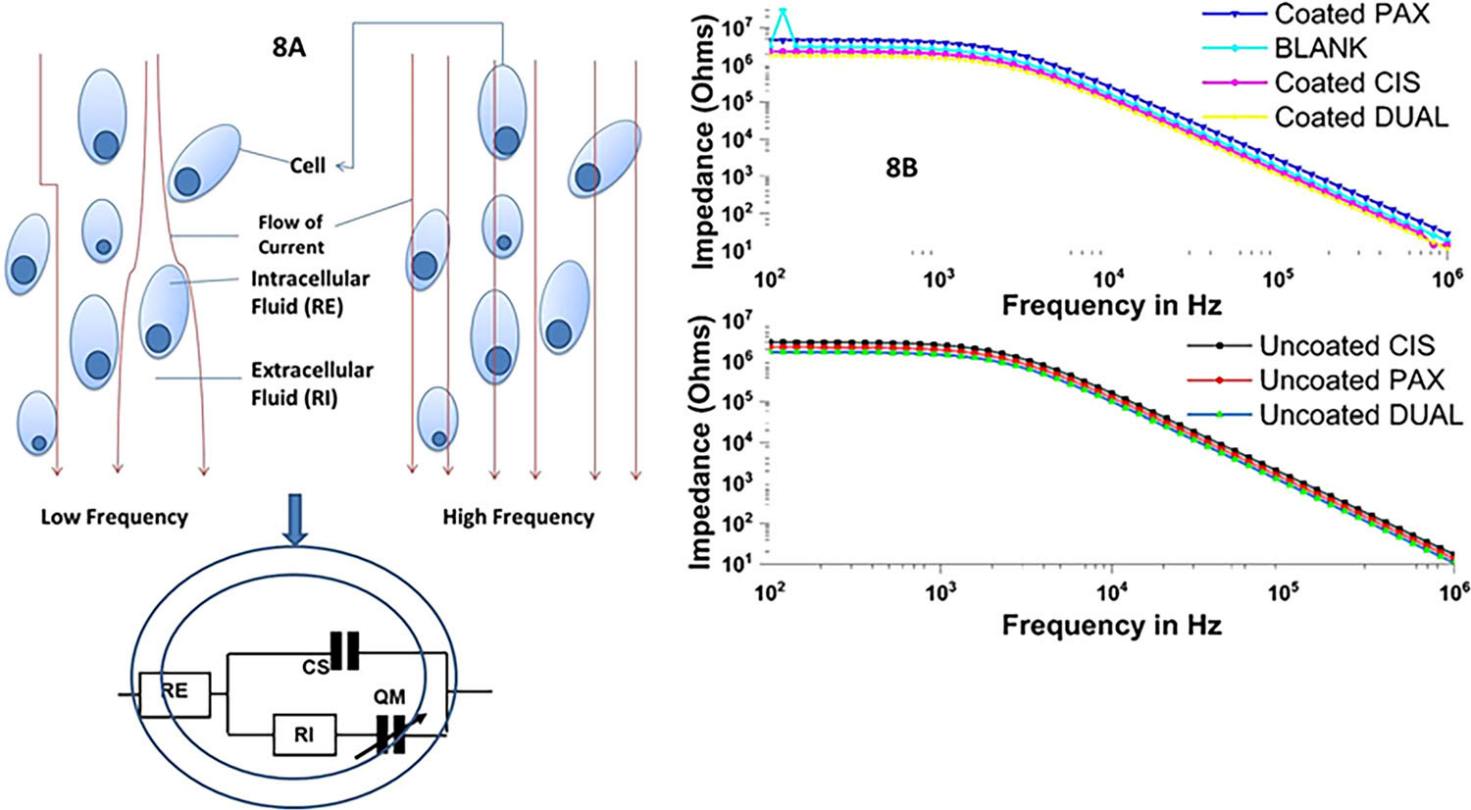
The device circuit of the biological cell. **a** Flow of current through cells in low and high frequencies and Cole–Cole model of biological cell. **b** Impedance measurement of cells treated with blank, coated and uncoated CIS, PAX, and DUAL cubosomes.

The ECIS device circuit of the biological cell was designed, as was adapted from a study by Pradhan et al.^36^ and is described in Fig. 8b. HeLa cells were chosen because of easier culturing methods and well documentation. We hypothesized that growth of the HeLa cell culture will lead to cellular metabolism, wherein the medium will consist of the by-products of the cellular metabolism which are extremely conductive in nature, thereby decreasing the impedance. Due to the incorporation of the cubosomal formulations, there are bound to be some changes in the biophysical signature of the cell culture. There must be some definite changes in the cellular impedance before and after treatment of cubosomal formulations. We can see clearly that with treatment of blank cubosomes, at lower frequency (100 Hz), the impedance of the cells is low. Blank cubosomes offer no resistance to the growth of HeLa cell cultures, and hence the low impedance. The same condition reapplies in the case of higher frequency also, where the cell impedance decrease because of the current passing directly through the cell membrane instead of channels, depicted in Fig. 8a. But upon treatment with uncoated cubosomes, the cellular impedance became very high because of death of large number of cells due to the initial burst release of the drug. However, it gradually dropped due to no drug being leaked out of cubosomes in a matter of time after 10 h. On the other hand, when treated with coated CIS, PAX, and DUAL cubosomes, the impedance had a steady high which then finally increased since no more drug leaked out after a slow and sustained release for 25 h. The best reduction in bioimpedance was caused due to coated DUAL cubosomes because of the synergistic effect obtained as a result of cisplatin and paclitaxel release simultaneously. Therefore, ECIS devices may also be used for diagnostic procedures to differentiate between treated and non-treated HeLa cells.

### In vitro fluorescent imaging of HeLa cells

**Fig. 9:**
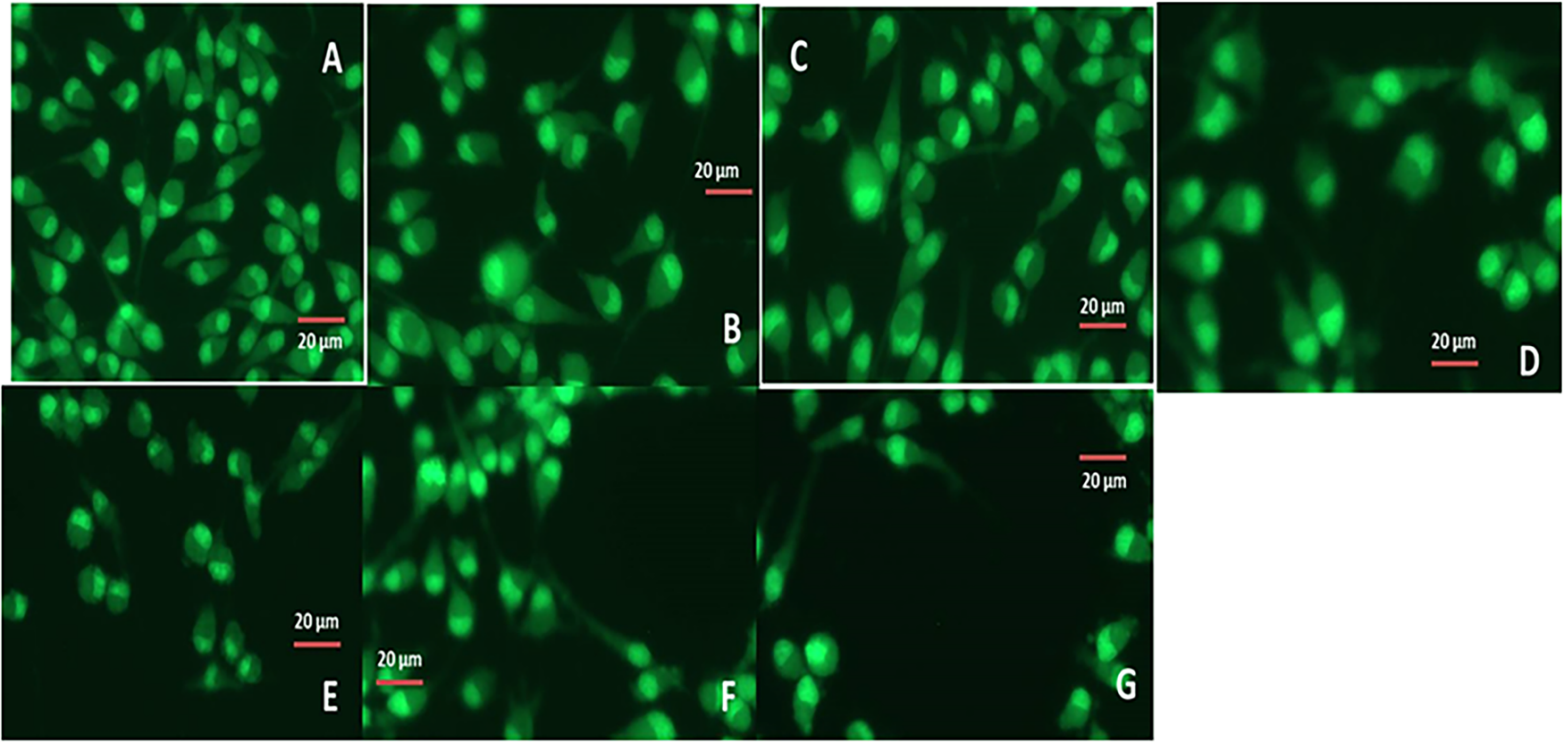
Fluorescent images of HeLa cells treated with blank, coated and uncoated CIS, PAX, and DUAL cubosomes.

Cell Explorer™ fluorescence imaging kits were used to stain the live HeLa cells. The HeLa cells act as an appropriate model for carcinoma tissue. This kit in particular is intended to equally mark all the live HeLa cells in green fluorescence. For this study in fluorescence microscopy, this was appropriate dye as the fluorescent docket molecules of the dye retained inside the HeLa cells for comparatively extended time period when compared with other dyes. The HeLa cells were fixed to keep the imaging array consistent. It is interesting to note that the dye is nonfluorescent before entering the cells, but potentially entraps a cell-retaining moiety. But immediately after entering the live cells, the dye becomes intensely fluorescent and confined within the live HeLa cells providing a consistent fluorescence signal for comparatively lengthier period of time. The dye consists of a hydrophobic composite, which easily infuses within intact live HeLa cells. The labeling process is an extremely robust one, as we can see in Fig. 9. The figure clearly illustrates that cells incorporated with blank cubosomes are unharmed, whereas the cell death is more in ones treated with coated CIS, PAX, and DUAL than the uncoated ones. This is because in the cells treated with uncoated cubosomes after the initial burst of drugs which causes cell death, release of drugs dropped down. This causes a regrowth of cells, whereas in the cells treated with coated cubosomes there is sustained release of drugs for 25 h which do not allow cell growth. Moreover in the DUAL-treated ones, due to the synergistic action of the two drugs, there is increased efficiency against the HeLa cells and minimum number of live cells are visible.

**Fig. 10:**
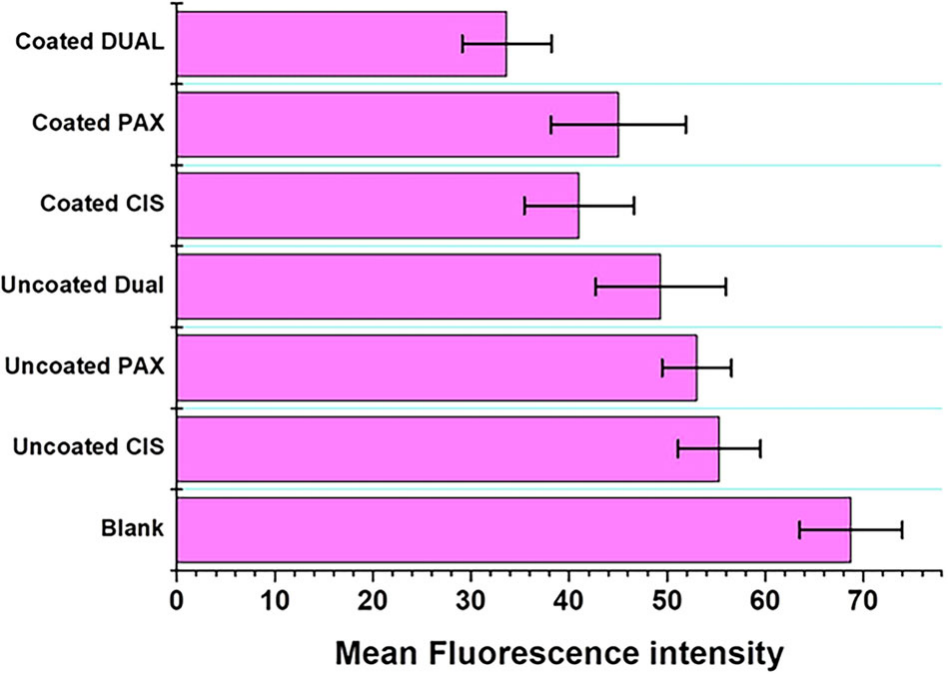
Mean fluorescent intensity of cells treated with blank, coated and uncoated CIS, PAX, and DUAL cubosomes.

In order to reinforce the results, determination of intensity of cellular fluorescence was done by ImageJ software depicted in Fig. 10. Control cells showed maximum fluorescent intensity (68.7206) followed by uncoated CIS, PAX, and DUAL cubosomes. This was due to initial burst release of drug and thereafter very nominal amount of drug release. The coated cubosomes released the drug over a period of 25 h, which restricted the growth of cells for longer period of time. Among them, coated DUAL cubosomes showed the least intensity (33.6521) due to combined/synergistic slow and sustained release of the drugs, cisplatin and paclitaxel. This gradual loss in the intensity of cellular fluorescence may be due to reduction in the metabolism or metabolic activities of the cell because of the exposure to drug-loaded cubosomes, which leads to disruption of cellular metabolism eventually leading to cell death.

## Discussion

Targeting therapy has become the new therapeutic approach for cancer treatment. This involves delivery of the anticancer drugs at the specific site without any cytotoxic effects on other tissues or organs^43^, where the effects of cancer may be reduced/reversed. The concept and development of lipid nanovehicles have been originated in 1970s, which are in the mould of continuous modification until today. An extremely recent development of this targeted cancer therapy is the lipid cubic crystalline nanovehicles, which are also termed as cubosomes. Cubosomes have garnered much interest due to the increased bioavailability of drugs at the target site. There have been extensive research on drug-loaded cubosomes at the cellular level, and few studies have reported in vivo administration too^1,21^. But to the best of our knowledge, there have not been much work done on loading the cubosomes with combinatorial drugs which may increase anticancer efficacy. Subsequently, there is not much literature to compare the cubosomes with that we have prepared in this study. But we compared our data wherever we could with the available pool of the data.

This study had been designed to take the cubosome research at the cellular level, a step further by loading the cubosomes with a combination of known and established anticancer drugs, cisplatin and paclitaxel. This study elaborates on the preparation and characterization of the dual drug-loaded cubosomes, which was extremely necessary before any in vitro study involving cell line was carried out. There have been reports that monooleins and poloxamer 407-based nanoparticles are not stable after drug loading, and immediately disperse after coming in contact with media^44^. Therefore, the concentration of poloxamer 407 which provides a steric stabilization to the cubosomes had been increased to 10% during preparation, as suggested by Nasr et al. The coating of poly-Ɛ-lysine was also targeted to provide a protective covering and prevent the initial burst release of drugs. The morphological analysis including size and zeta-potential measurement, DSC and XRD studies have clearly indicated the uniform dispersion of drug in the cubosomes. Human hepatoma HepG2 cell line was chosen for cytotoxicity studies, because their entire surface area has been reported to be available for these nanovehicles as opposed to HeLa cells where only a partial area is available. Hence, this cell line is one of the most sensitive^45^, and the mechanism and the effect of cytotoxicity may be more pronouncedly understood. The cytotoxicity results suggest coated cubosomes have lower toxicity compared with uncoated ones, but it is also evident that there is a significant difference between the cytotoxicity of coated and uncoated cubosomes. This is an interesting find since there are reports that cubosomes have higher toxicity than monooleins combined with drug^46,47^. Therefore, it was highlighted by our study that coating may reduce the cytotoxicity of the cubosomes too. The entrapment efficiency and in vitro release studies emphasize the importance of poly-Ɛ-lysine coating on the cubosomes for a slow and sustained release of the drug which had also been previously noted by Deshpande et al.^12^.

In both the in vitro cellular studies (impedance and fluorescent imaging), HeLa was the chosen cell line because of two reasons; HeLa cells are more resistant to toxicity of these nanocarriers; hence the mechanism of action of these nanocarriers may be easier to elucidate, and HeLa cell are also less adherent to these nanocarriers than normal cells. As is elaborated in the data, the coated cubosomes are more effective in destruction of HeLa cells because of their slow and sustained release as against the burst release of uncoated ones. It may be interesting to note that despite the two reasons cited above, there is increased destruction of HeLa cells which proves the efficiency of the CIS-, PAX-, and DUAL-coated cubosomes in targeting cancer cells.

This study underlines the importance of cubosomes as an efficient nano drug carrier, especially for the hydrophobic drugs. This is in line with the various other studies where cubosomes acted as nanovehicles for anticancer drugs^25,27,46^. This study may also act as guide to design combinatorial drug-loaded coated cubosomes for therapeutic approaches toward cancer.

## Conclusion

Cubosomes are nanostructures that are self-assembled in a unique association, primarily made up of monoglycerides, which in our case was RYLO, composed of monooleins. In our study, these are prepared by top–down approach which depicted to produce cubosomes with little aggregation. Cubosomes prepared are shown to be have added benefit of large surface area, where even the poorly soluble hydrophobic drugs are dispersed uniformly losing their crystallinity. Coating cubosomes result in preventing the initial burst release of drug, thereby providing a slow and sustained release. Before the cubosomes may be adapted as nanocarriers for drug delivery, the only concern is the improvement of the adherence of drugs to the lipid core bisected by a lot of aqueous channels, which otherwise promotes drug leak. In vivo studies are also required to actually understand the efficacy of the cubosomes in treating carcinoma.
